# Circumventing the Impossible: Cell-Free Synthesis of Protein Toxins for Medical and Diagnostic Applications

**DOI:** 10.3390/ijms252413293

**Published:** 2024-12-11

**Authors:** Alina Mai Woelbern, Franziska Ramm

**Affiliations:** Fraunhofer Institute for Cell Therapy and Immunology, Branch Bioanalytics and Bioprocesses (IZI-BB), Am Mühlenberg 13, 14476 Potsdam, Germany

**Keywords:** cell-free protein synthesis, toxins, in vitro translation, venoms

## Abstract

Naturally occurring protein toxins can derive from bacteria, fungi, plants, and animal venom. Traditionally, toxins are known for their destructive effects on host cells. Despite, and sometimes even because of, these harmful effects, toxins have been used for medical benefits. The prerequisite for the development of toxin-based medications or treatments against toxins is thorough knowledge about the toxin and its underlying mechanism of action. Thus, the toxin of interest must be synthesized. Traditional cell-based production requires high laboratory safety standards and often results in a low total protein yield due to the toxin’s harmful, cytotoxic nature. These drawbacks can be circumvented by using cell-free protein synthesis (CFPS), a highly adaptable platform technology relying on cell lysates rather than living cells. This review discusses the current advances in cell-free synthesis of protein toxins as well as their uses and applications for pharmaceutical and diagnostic purposes.

## 1. Introduction

Toxicity can be described as the harmful consequence of a cell’s or organism’s exposition to a toxin. A toxin is a substance that induces pathophysiological reactions in the host in a dose-dependent manner. Thus, a toxin causes damage to living beings, causing the inhibition of cellular pathways or even cell death [1]. As per definition by the late Paracelsus: the dose makes the poison [2]. With that in mind, it can be stated that nature itself produces the most efficient toxins. A large class of naturally occurring toxins are protein-based compounds from bacteria, fungi, plants, and animals. Nature has ubiquitously provided proteins for infection, self-defense mechanisms, or to harm or even kill prey [3,4]. The main aim of toxicology is therefore to identify the modes of action of toxins and subsequently develop inhibitors or blockers. Coming back to Paracelsus’ toxicity concept, Paul Ehrlich has developed the magic bullet concept, in which toxic compounds might be utilized for a medical benefit, such as for cancer therapeutics [5]. 

Unfortunately, experimental settings for research and development regarding such toxins require the pure protein toxin. Recombinant protein production includes a variety of chemicals, supplements, and cellular compartments or even whole cells. Thus, before its application, the protein has to be purified from a mixture consisting of the protein itself and the synthesis components, and in cell-based expression systems, the protein has to be extracted from the cell or inclusion bodies. This is vital for the identification of the specific functionality of the protein and might even be necessary to retrieve the protein of interest from endotoxins within the protein production system [6]. Especially considering toxin synthesis, producing organisms can endogenously express toxins. The purity of recombinant toxins is necessary to specifically assess the mode of action of protein toxins, as well as for the synthesis of protein toxins as reference material and calibrants for assays [7]. Moreover, when considering the recombinant production of biopharmaceuticals, there are strict guidelines for the purity of the product [8]. These facts also hold true when studying protein toxins. 

The toxins can either be extracted directly from the naturally producing organism or need to be expressed synthetically. Bacterial toxins can be purified from their naturally producing organism or be synthetically overexpressed in bacterial strains. In 2004, Lindbäck and colleagues showed that two subunits of the tripartite toxin Non-hemolytic Enterotoxin (Nhe) could directly be purified from *Bacillus cereus* culture supernatants, and the recombinant expression of each individual subunit was possible in diverse bacterial strains [9]. Unfortunately, the generation of these toxin-producing strains oftentimes leads to higher laboratory safety standards. The synthesis of protein toxins in eukaryotic cell-based systems faces the same challenge. On top of that, various toxins are difficult-to-express proteins, as they target eukaryotic cells, their compartments, and biochemical reactions and pathways such as ribosomes and protein translation [10]. Animal toxins usually occur in venoms, which can be acquired from the venomous glands by applying pressure or electric stimulation [11,12].

An alternative to circumvent these drawbacks is cell-free protein synthesis (CFPS).

**Figure 1:**
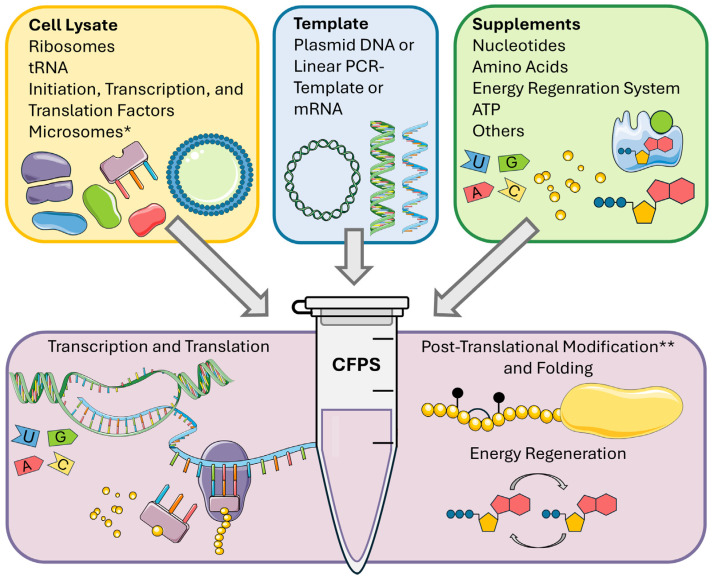
Principle and basic components of cell-free protein synthesis (CFPS). * Microsomes are found only in specialized eukaryotic lysates. ** Possible post-translational modifications vary between lysates. PCR: Polymerase chain reaction. Designed and modified with https://smart.servier.com/ (accessed on 27 August 2024).

CFPS, also known as in vitro protein synthesis, relies on cell lysate rather than living cells. Translationally, active lysates are prepared from raw cell extracts by removal of cell membranes and nuclei, as well as digestion of endogenous nucleic acids [13,14,15]. The resulting lysates contain all essential components for protein translation, including ribosomes, tRNAs, and translation factors [16]. To facilitate protein synthesis, the system is supplemented with amino acids, NTPs, ATP, and an energy regeneration system in the presence of a circular or linear template for the protein of interest [13,17,18]. Furthermore, the supplementation of salts, such as magnesium, potassium, or sodium salts, can be detrimental for lysate preparation as well as for the synthesis reaction [19,20,21] (Figure 1). 

The open nature of the cell-free system allows a variety of adaptations based on the individual needs of the protein of interest, such as the addition of chaperones [22]. Protein translation can be carried out in parallel to transcription (coupled mode) or separately (linked mode) [13,23]. For the linked mode, purified and processed mRNA is added to the cell-free system [24], whereas the coupled mode uses a DNA template. The cell lysates used for CFPS are prepared from a variety of cell types, including prokaryotic, plant, fungal, insect, and mammalian cells [25]. To date, *E. coli* lysate has been considered to produce the highest yields, but it catalyzes only a limited number of post-translational modifications (PTMs) and lacks membranes, impeding the synthesis of membrane proteins [25,26]. Eukaryotic lysates, on the other hand, can facilitate a variety of PTMs, including glycosylation, disulfide bonding, and phosphorylation [27,28,29,30]. This is due to the presence of microsomes, membranous vesicles derived from the endoplasmic reticulum (ER) [13,31]. The microsomal lumen provides a redox potential, facilitating disulfide-bridge formation and chaperones and aiding the correct formation of tertiary structures [27,32]. Additionally, synthesized membrane proteins can be integrated into the microsomal membranes [20,29,33]. 

Cell-free systems were originally used to study the translation machinery and proteins inhibiting translation [24,34,35], but the focus has since switched to the characterization and analysis of proteins up to production for downstream applications [24]. Because cell-free systems are easily adaptable and can synthesize proteins within a few hours, they present an opportunity for high-throughput screening of proteins. This has been exploited to produce proteinaceous drugs (e.g., antibodies, hormones, enzymes) or drug targets (e.g., cancer epitopes) for research purposes [36,37,38,39]. CFPS has also been used as a convenient tool for the inclusion of noncanonical amino acids (ncAA), which is achieved by amber stop codon suppression and supplementation of a modified tRNA and aminoacyl-tRNA synthetase pair [40]. This extension of the genetic code has proven useful for the synthesis of antibody–drug conjugates (ADCs) [41,42]. Furthermore, difficult-to-express proteins such as membrane proteins, viral proteins, and toxins have been produced in cell-free systems [20,33]. Toxins, which are difficult to express in viable cells because of their inherent toxicity, can easily be translated in cell-free systems. Moreover, the generation of genetically modified organisms producing toxins can be circumvented by using PCR products as a template for protein synthesis, thus lowering safety regulations [43].

## 2. Cell-Free Synthesis of Toxins

Since the development of cell-free systems in the 1960s [24], a range of different protein toxins have been synthesized [44,45]. Table 1 summarizes the current research articles studying protein toxin synthesis (omitting cytotoxic viral proteins) in cell-free systems and includes the cell-free system used for the synthesis as well as the subsequent downstream application and functional activity. Unfortunately, the functional activity of the synthesized toxins was not studied in all research articles.

### 2.1. Bacterial Toxins

While most bacteria are harmless or even beneficial for humans (e.g., bacteria in the gut flora), some bacterial infections can cause a wide range of pathophysiological effects and disease patterns [3]. Such infections can affect the gastrointestinal tract and lead to fever or pulmonary diseases [69,70]. One reason for this is the diversity of bacterial strains and the different toxins they produce. There are numerous classes of toxins, such as superantigens and neurotoxins, with severe pathophysiologies, but also diverse entero- and exotoxins with milder symptoms.

Superantigens and neurotoxins from bacterial strains can lead to more severe disease patterns. Superantigens such as those from *Staphylococcus aureus* can trigger intense immune reactions by activating the MHC II/T cell receptor complex, which can lead to the toxic shock syndrome [71]. Bacterial neurotoxins such as the botulinum neurotoxin or the tetanus neurotoxin, target neuronal structures, leading to neurological disorders and pathophysiological consequences such as necrotic foci of the nervous tissue [72].

The class of heat-stable enterotoxins is mainly responsible for infections of the gastrointestinal tract, which could lead to travelers’ diarrhea derived from enterotoxigenic *E. coli* [73]. Bacterial exotoxins often consist of multiple subunits or multimers, making them difficult to express. This is exemplified by two main groups of bacterial toxins: pore-forming toxins (PFTs) and AB_5_ toxins.

PFTs are highly diverse and constitute the largest group of bacterial toxins [74]. The transmembrane regions of PTFs consist of either α-helices or amphipathic β-strands, and the group is divided accordingly into α- and β-PFTs [75]. For both groups, the first step towards pore formation is the binding of toxin monomers to receptors on the surface of target cells. In α-PFT monomers, receptor binding triggers a conformational change, exposing hydrophobic residues and facilitating concomitant membrane insertion and oligomerization [76,77]. β-PFTs, on the other hand, form a pre-pore before membrane insertion [74,78,79]. Both types of pores expose hydrophilic residues in their lumen, allowing the uncontrolled influx and efflux of ions, proteins, and nutrients.

Another large group of bacterial exotoxins are AB and AB_5_ toxins, which are named after their two distinct subunits [80,81]. As the name suggests, they contain a single A (catalytically active) subunit and one or a ring of five B (receptor binding) subunits. A well-known representative of the AB toxin family is the diphtheria toxin, in which the catalytic A subunit is an ADP-ribosyl transferase [81]. AB_5_ toxins are further distinguished by their catalytic activity. The pertussis toxin (Ptx) and cholera toxin (Ctx) groups catalyze the ADP-ribosylation of G_s_α and G_i_α proteins, interfering with G-protein signaling and resulting in an increase in intracellular cAMP. This leads to a dysregulation of the ion transport and is the cause of several foodborne diseases [82]. Toxins from the smaller Shiga toxin (Stx) group target ribosomal RNA, inhibiting eukaryotic translation. Lastly, subtilase toxin (SubAB) cleaves binding immunoglobulin protein, a 70 kilodalton heat shock protein (HSP70) chaperone residing in the ER [83]. The A peptide of AB_5_ toxins contains the catalytically active A1 domain and is inserted into the B pentamer through the A2 domain. Both domains are connected though disulfide bonds. After attaching to a target cell, the toxins are transported to the ER through receptor-mediated endocytosis [84]. There, the A peptide is cleaved by a bacterial protease and disassembled through a protein disulfide-isomerase from the host cell, releasing the active A1 domain [85,86].

#### 2.1.1. Synthesis of Multimeric Toxins with Distinct Subunits

PFTs have been studied more thoroughly in cell-free systems. Several studies have demonstrated the synthesizability of functionally active PFTs. α-hemolysin expressed in an *E. coli* cell-free system formed pores bilayers as visualized by microscopy [48]. Hemolytic activity, cell-based toxicity, and electrophysiological characterization are the assessments for functionality when regarding PFTs. In 2001, the first bacterial PFT was synthesized in cell-free manner and evaluated for its functionality. The synthesis of leukocidin in a prokaryotic cell-free system helped to elucidate its structural and functional characteristics, identifying large ionic channel formations not found in α-hemolysin, which partially shares sequence identity [47]. This demonstrated the necessity for cell-free systems for the analysis of PFTs, as the fast study of different pores could help understand the mode of action of the protein toxin.

The highly heterologous group of PFTs comprises proteins with a single protomer, but also bi- and tripartite toxins. For bi- and tripartite PFTs, the ratio of the different subunits can be essential for pore formation. An example of this is the Nhe originating from *B. cereus*. In a cell-based study, the different subunits of Nhe were expressed separately in *E. coli* and mixed in different ratios before their inhibition of protein synthesis in Vero cells was studied. The maximal inhibition in the linear range of 30–70% was observed when the subunits were mixed in a 10:10:1 molar ratio of NheA–NheB–NheC [9]. Achieving this ratio of protein subunits in living cells is difficult because of the lack of control over the quantity of overexpressed gene products, as well as differing transformation efficiencies. In cell-free reactions, the open nature of the system allows easy manipulation of the synthesis reactions, as multiple templates can simply be added to the reaction at defined DNA/RNA concentrations. Using a CHO-based cell-free system, the previous results could be refined [52]. CFPS reactions with different template ratios showed that an excess of NheA and NheB over NheC is generally necessary for pore formation, with the highest ratio of templates allowing oligomerization being 10:10:2. Furthermore, the ratio of protein subunits was calculated to be 11.6:7.2:2.2 if a template ratio of 10:10:1 was used. Similar experiments were performed with the closely related hemolysin BL (Hbl) from *B. cereus* [53,87]. In this case, the ratio of the three subunits, the lytic domains L1 and L2 and the binding domain B, did not influence the hemolytic activity of the toxin [53]. Furthermore, both coexpression of all subunits and mixing of soluble protein after separate expression yielded functional Hbl pores. The effect of cell-free synthesized Hbl and its subunits on the plasma membrane integrity of CaCo2 cells was studied with a propidium iodide uptake assay. Interestingly, the L2 subunit alone caused an increased propidium iodide uptake, and coexpressed L1 and L2 subunits led to an uptake surpassing that of the holotoxin. These results suggest the formation of a pre-pore in the cell membrane. 

These studies demonstrate the feasibility of the synthesis and characterization of PFTs in cell-free systems. CFPS led to an increased understanding of the toxins’ mode of action and showed that homo- as well as multimeric toxins could be synthesized. In one of the first studies synthesizing a protein toxin in a cell-free manner, the diphtheria toxin from the AB toxin class was successfully synthesized in *E. coli* extracts in 1974. Phage DNA was isolated and directly applied for cell-free synthesis, leading to a functionally active protein toxin without the need of further optimization [44]. AB or even AB_5_ toxins generally reflect a different mechanism for complex formation, indicating the necessity of different synthesis parameters. Traditionally, the protein ratio is determined for complex formation. Even though no data on the effects of varying molarities of templates for the synthesis of functional AB_5_ toxins has been published, successful cell-free reactions used ratios of 1:1:5. (A1–A2–B) or 1:5 (A–B) [55,57]. To elucidate the possibility of an optimal subunit ratio, the parallel synthesis of A and B monomers is a more suitable approach compared with post-translational mixing, because simultaneous translation is a prerequisite for the formation of some AB_5_ holotoxins. This is due to the required disulfide bridging, which is achieved in the microsomes of eukaryotic cell-free systems [55,57].

#### 2.1.2. Bacterial Toxins Inhibiting Protein Synthesis

Toxins inhibiting the transcription or translation are especially difficult to synthesize, even in cell-free reactions. Shiga toxin (Stx), an AB_5_ toxin derived from *Shigella dysenteriae* or distinct *E. coli* strains, depurinates a conserved adenine residue in the 28S rRNA of the 60S ribosomal subunit, thus acting as a ribosome-inactivating protein (RIP) [88]. Using CFPS, Stx could be synthesized in both *E. coli* and CHO lysates [57]. To enable the synthesis of an RIP specifically inhibiting eukaryotic translation in a eukaryotic cell-free system, Stx subunits were translocated into microsomal vesicles, separating the toxin from the ribosomes. This was achieved by expression of the monomers as fusion proteins with a melittin signal sequence [21]. Additionally, a dialysis system (continuous exchange cell-free, CECF, system) was used. In the CECF system, a membrane separates the reaction chamber from a second chamber filled with feeding buffer. This setup allows a prolonged reaction and thus increases the overall protein yield [21]. Interestingly, higher yields were achieved with the *E. coli* lysate, but multimerization of B subunits was observed only in the CHO-based reaction. Both systems were able to produce active Stx, as was shown by cell-free and cell-based inhibition of protein translation. In a cell-free reaction synthesizing the model protein luciferase, the addition of Stx led to a decreased protein synthesis rate. Additionally, the inhibition of protein synthesis in HeLa cells was demonstrated.

Stx and Shiga-like toxins are two of only a few RIPs originating from bacteria [89,90]. Typically, plant-based toxins such as ricin come to mind. Throughout the last decades, cell-free systems have been used to study the inhibitory effect of RIPs on the synthesis of model proteins. Numerous studies have added isolated RIPs to cell-free systems, demonstrating one application of CFPS. As a rapid approach to screen the activity of RIPs, different cell-free systems can be used to compare their effects on prokaryotic and eukaryotic ribosomes [91,92,93].

#### 2.1.3. Modifications and Applications of Bacterial Toxins

Toxins derived from cell-free reactions have been used for a variety of experiments. Various studies have tested the synthesizability of PFTs in cell-free systems and applied diverse methodologies for their functional assessment. As the pore-forming character is essential for the toxicity of the protein, most studies have focused on electrophysiological analyses based on pore formation in planar lipid bilayers. Classical cell-based toxicity assessments have been performed in CaCo2 cells, showing the toxicity of two PFTs from *Bacillus cereus* [52,53]. To test the combined pore-forming and toxic properties of PFTs, hemolysis assays can be performed for most PFTs. Such assays can be performed using purified erythrocytes or blood agar plates using blood from sheep, bovines, or rabbits [47,49,53]. In comparison with cell-based protein expression, a major advantage of CFPS is that the synthesized proteins can be tested on living cells without further purification steps, as they are not contained within the cell. Additionally, many bacteria contain endotoxins, which influence the results of cell-based assays. In contrast to cell-based protein synthesis, the reaction conditions are highly variable and adaptable to the protein’s need. This also includes the vessel in which the reaction is performed. This was exemplified with α-hemolysin (αHL), a β-PFT. A fusion protein of an αHL monomer and enhanced green fluorescent protein (eGFP) was synthesized inside of large phospholipid vesicles [48]. This was made possible through a transcriptional activation cascade delaying the start of transcription by approximately 30 min, allowing the inclusion of the CFPS components into the vesicles. The reaction was monitored by visualization of eGFP fluorescence. αHL pores were formed in the lipid membrane, allowing a selective feeding of nutrients into the lumen. After pore formation, the system thus worked identically to a CECF system, prolonging the reaction time and increasing the protein yield. Additionally, the interaction of αHL-eGFP with a supported lipid bilayer (SLB) was studied quantitatively in real time. For this, cell-free reactions synthesizing eGFP or αHL-eGFP were injected into a quartz crystal microbalance–dissipation (QCM-D) device containing a SLB. Briefly, QMC-D uses a highly sensitive sensor detecting mass changes at its surface. The device applies voltage to a piezoelectric quartz sensor and measures its mass-dependent oscillations [94]. Changes in the crystals’ oscillation frequency provided real-time data on the integration of αHL pores into the SLB. The formation of stable membrane pores was detected 0.5 to 1 h after injection and confirmed by atomic force microscopy. This method allows studying the interaction of toxins with different types of membranes or membrane proteins, which helps to understand the mode of action of the toxin.

Several analytical methods for studying toxins and their modes of action require the modification of toxins. However, changes in the polypeptide sequence, as well as fusion or conjugation to other molecules, can disturb protein folding, oligomerization, or the toxin’s function. For this reason, the precise locations and numbers of ‘tags’ need to be optimized. This is facilitated by the open nature of CFPS, which allows comparatively quick screening of different modifications. A variety of possible modifications were demonstrated using the AB_5_ toxins heat-labile enterotoxin (LT) and Ctx [55]. Both were synthesized in CHO and *Sf*21 lysates. It was demonstrated that both the A and B subunits could be modified. A fusion of LTB to streptavidin and subsequent coupling to a biotin-conjugated fluorophore was possible without disrupting multimerization. Furthermore, ncAAs could be inserted within the catalytic center of CtxA to allow its fluorescent labelling. In contrast to the unmutated holotoxin, the mutated constructs did not show any cytotoxic effects on CHO-K1 cells. This was likely due to the incorporation of the ncAA in the catalytic center. This ‘silencing’ and fluorescent labelling of the toxins facilitates their use for intracellular tracking studies, as they will not kill target cells.

As these studies have shown, adding tags or fluorescent proteins to toxins and even integrating mutations is easily feasible within cell-free systems. Especially considering multimeric toxins, the modification of the N- and C-terminus can affect their complex formation and thus the functional activity of the toxins. Bechlars et al. designed fusion constructs of thermostable direct hemolysin (TDH) with purification tags by polymerase chain reaction (PCR). The results implied, on one hand, that a parallel screening of different DNA templates and subsequent testing of the functional activity in a parallelized manner is possible in cell-free systems. On the other hand, the data showed that the addition of tags can hinder the toxicity or functional activity and thus demonstrated the necessity of a prescreening [49]. In a doctoral thesis, defined mutations were inserted into the coding sequence of the PFT cytotoxin K (CytK) from *B. cereus*. Within a CHO-based cell-free system, the changes in the coding sequence led to a different multimerization pattern of this heptameric toxin, resulting in different activities [56]. CFPS can be used to study mutations that might occur in the future by applying directed evolution techniques in a fast and efficient manner, as the adaptation of the template can be performed in a PCR reaction, eliminating the need for cloning procedures while allowing high-throughput screening. PCR template generation can also be used to synthesize toxin fragments and potentially identify active centers/toxic domains. Zichel and colleagues used an *E. coli*-based cell-free system to synthesize the heavy chain fragment from the neurotoxin botulinum toxin and tested these fragments as potential vaccine candidates by immunizing mice. After the immunization, a serum antibody titer could be detected, and mice were protected against the toxin challenge [95]. This reflects the medical use of CFPS and demonstrates CFPS as an alternative methodology for the generation of vaccine candidates.

**Figure 2:**
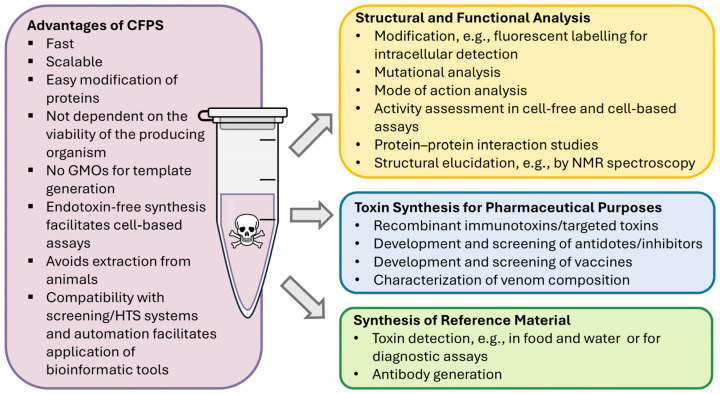
Downstream applications of cell-free toxin synthesis. GMO: genetically modified organism; HTS: high-throughput screening; NMR: nuclear magnetic resonance.

The data acquired for bacterial protein toxins reflects the diversity of the applications that are facilitated by using cell-free systems, as summarized in Figure 2. 

### 2.2. Plant-Based Toxins

Plants express toxic proteins as a means of protection against predators such as pathogens, insects, or other living organisms. These toxins can ultimately help the plant to survive and continue growing. Humans can be exposed to plant-based toxins by physical contact or ingestion of plants or plant-based foods and medications. 

The variety of toxins expressed in plants is very diverse, ranging from RIPs and PFTs to ureases, alkaloids, and lectins [96,97]. These toxins can cause minor illnesses, mainly affecting the gastrointestinal tract and resulting in vomiting or diarrhea, but they can also cause serious pathophysiological conditions leading to heart failure or death.

To our knowledge, there has been no published study on the synthesis of proteinaceous plant toxins in cell-free systems. Nevertheless, as some of the protein classes that are associated with toxicity are widely known, these studies might not have classified these proteins as toxins. A doctoral thesis aimed to synthesize the RIP Dianthin from the ornamental plant *Dianthus caryophyllus* L. (commonly known as clove pink). In an approach like those shown for bacterial RIPs, Dianthin was synthesized in a CHO-based cell-free system, enabling the cotranslational translocation of Dianthin into microsomes by using a melittin signal peptide. Dianthin was analyzed on its own or as a fusion protein with an epidermal growth factor, facilitating the development of a targeted toxin. Within cell-based toxicity assays, as well as an in vitro protein translation inhibition assay, both the single protein and the fusion protein were functionally active [56]. This thesis demonstrated the feasibility of the synthesis of plant-based RIPs in cell-free systems. Nonetheless, the class of plant toxins demonstrates a vast variety of toxins that should be synthesized in cell-free systems to efficiently validate CFPS as a tool for the synthesis, characterization, and application of plant toxins.

Similarly, as in the analysis of bacterial RIPs, plant-based RIPs have been evaluated for their ribosome-inactivating character in eukaryotic cell-free systems [98]. This further exemplifies the diversity of application of CFPS for protein toxin research.

### 2.3. Animal Toxins

Animal toxins can majorly be found in venoms. Venoms include a combined mixture of different specialized toxins and other proteins and peptides that are produced by animals. These mixtures are directly delivered into the tissue of the prey by fangs or tails with venom glands. Venoms and their cocktails of different proteins reflect the high biodiversity of different protein toxins, underlining the necessity to have a platform to analyze the characteristics of venoms. It is essential to understand the mode of action of each individual toxin within venom and the interplay of all venom components. Furthermore, the analysis of venom can lead to the development of novel biopharmaceuticals. In the past, research identified that peptides from venom can be used as compounds for the treatment of cardiovascular diseases. Captopril, for the treatment of hypertension, is based on a peptide from a snake venom [99,100].

#### 2.3.1. Complexity of Venom Protein Synthesis

The potential of cell-free systems to analyze the components of venoms was already detected in the 1970s [45,59]. Diverse studies used cell-free systems to analyze the mRNA of venom. The synthesizability of the acquired mRNA was tested, and subsequently, the proteins could be analyzed. In a study by Suchanek and colleagues, the mRNA of the pore-forming toxin melittin from honeybee venom was translated. Interestingly, instead of a traditional cell lysate, this work used the separate components of mouse liver ribosomes, rat liver enzymes, and initiation factors from rabbit reticulocytes, marking a first study to express a venom protein in a mammalian system [59]. Later studies analyzed the cell-free synthesis of bee venom proteins in wheat germ lysate [45,59,60]. The proteins could be synthesized, and protein sequencing could be performed to gather novel insights into the composition of these venom protein toxins. In 1985, a similar approach was performed in wheat germ as well as rabbit reticulocyte lysates. The mRNA from the venom glands of the South African puff adder snake was translated, and the resulting proteins were analyzed for signal peptides and the activation of antiserum [61]. Within these studies, the mRNA encoding the venom proteins was extracted from the animals. Later studies have used DNA templates encoding the respective proteins. In one study, the synthesis of U_2_-sicaritoxin-Sdo1a from the venom of the six-eyed sand spider *Hexophtalma dolichocephala* was attempted in three different lysates. The toxin could be synthesized using the *E. coli* PURE system, but not the general *E. coli* or the *Sf*21 system. U_2_-sicaritoxin-Sdo1a is an inhibitory cysteine knot motif (knottin) harboring complex disulfide-bridge structures. If the *E. coli* and *Sf*21 systems had been unable to build these complexes, the synthesis of active protein might have been hindered [66]. Wang and colleagues showed that optimization of the cell-free system can increase the total protein yield of the synthesized venom protein. The protein of interest was venom kallikrein, a serine protease with potentially therapeutic characteristics in the cardiovascular field. The optimizations of the CFPS resulted in an increased specific enzyme activity. Nonetheless, in comparison with kallikrein from the crude venom extract, the synthetic protein showed an activity of 1.3 U/mg as compared with 1.74 U/mg [62]. Furthermore, a metalloproteinase from *Bothrops jararaca* snake venom was synthesized in *E. coli* lysate. The synthesized proteins were shown to be functionally active and efficiently bound to target molecules [65]. A large study in 2024 compared the synthesis of 30 different proteinaceous components of venoms. Using an *E. coli* PURE system, only 4 out of 30 tested proteins could be synthesized. Unfortunately, the functional activity of the proteins was not tested [67].

These studies demonstrate that it is essential to adjust the cell-free system to the needs of individual venom proteins. As venoms are diverse in their composition, the proteins vary tremendously. An individual setup for each protein class is necessary. Complex PTM structures and folding mechanisms are necessary to ensure the synthesizability of the proteins and their functional activity. It appears that a one-fits-all cell-free system solution is not possible for venom compositions. Nonetheless, single venom proteins and peptides can be synthesized and might be used as reference materials to produce antitoxins/antisera and to test inhibitors against venom proteins. Furthermore, the last century has shown that proteins from venom can be immensely useful as biopharmaceuticals, e.g., in cardiovascular research [101]. Hence, CFPS can be used to screen diverse protein sequences for the development of novel venom-based pharmaceuticals.

#### 2.3.2. Novel Approaches in Venom Protein Research

Bioinformatic tools, as well as artificial-intelligence-based tools, have modernized the scientific world. CFPS has been combined with design of experiments, automated processes, and prediction models [102,103,104]. 

In 2023, immunoinformatics were coupled with the cell-free synthesis of the cytotoxin from cobra venom. Epitope sequences were characterized using immunoinformatic tools and molecular docking simulations for improved antivenom development. Mutagenesis of the template sequence was performed based on the prediction, and the plasmid DNA was transcribed. At last, the proteins were expressed in an *E. coli* system and subsequently purified. The cytotoxicities of the wild type and mutants were compared in a HaCaT cell-based assay. Defined epitope sequences, which are relevant for structural folding and the peptides cytotoxicity, were identified [105].

This study not only combined bioinformatic approaches to enhance the analytical throughput but integrated mutational analyses and screening technology, demonstrating the interdisciplinary approach of cell-free toxin synthesis.

### 2.4. Toxins as Pharmaceuticals: Targeted Toxins

Targeted toxins, and specifically recombinant immunotoxins (RITs), are chimeric molecules designed to target and kill specific cell types. They consist of a targeting moiety such as an antibody or antibody fragment, an optional linker, and a proteinaceous toxic moiety. Unlike ADCs, for which a (synthetic) drug is conjugated to an antibody post-translationally, RITs can be synthesized in one step, as they consist of a single polypeptide chain [106,107]. Toxins that have been used for that matter include bacterial toxins catalyzing the ADP-ribosylation of eukaryotic elongation factor 2 (eF2), such as Pseudomonas exotoxin A (PE) and diphtheria toxin (DT). Both toxins contain an enzymatic, a cell binding, and a membrane translocation domain. Plant-derived toxins such as ricin and gelonin, which target eukaryotic 28S ribosomal RNA, have also been investigated for their potential use as a toxic moiety [108]. As mentioned before, synthesis of these toxins is difficult in eukaryotic cells. Instead, the toxins are typically produced in *E. coli*. This is also the case for the three FDA-approved targeted toxins/RITs, denileukin diftitox (DT-IL-2, Ontak®) [109], tagraxofusp-erzs (DT-IL-3, Elzonris®) [110], and moxetumomab pasudotox (anti-CD22 dsFv-PE38, Lumoxiti®) [111]. A major advantage of CFPS is the possibility of downscaling the synthesis reactions. As CFPS allows high-throughput parallel synthesis, and as there is no need for refolding and purification after protein harvesting from inclusion bodies, screening of a larger variety of candidate proteins is facilitated. This is crucial, because proteinaceous drugs that are partially derived from nonhuman organisms need to undergo several steps of optimization, for example, the removal of T-cell epitopes [112]. Nonetheless, only two RITs have been synthesized in cell-free systems so far.

The first cell-free synthesis of a RIT was performed in 1993. Rabbit reticulocyte lysate was used in a linked mode to synthesize RITs based on a single-chain variable fragment (scFv). The RITs targeted the human transferrin receptor (TfnR), a cell-surface receptor that is predominantly expressed in carcinomas and sarcoma. The toxic moieties used were two DT mutants (CRM 107 [S(525)F] and DTM1) and a truncated but enzymatically active version of PE (PE40). In a cell-based protein translation inhibition assay, all RITs were able to completely suppress ^14^C-leucine integration during protein synthesis in K562 cells [68]. A second study in 2022 used PE24, the shortest currently known version of PE with the capacity to kill cells, as a toxin. It was fused to CD7, a receptor that is expressed on T- and NK cells and associated with graft vs. host disease and several types of leukemia. Lysates derived from *E. coli* and CHO cells were used to synthesize the RIT in a coupled transcription–translation reaction. Again, the RIT was translocated into microsomes in the CHO lysate to separate the toxins from the ribosomes. The *E. coli*-based reaction was equipped with chaperones, either by supplementing or transformation with the according plasmids prior to lysate production. As expected, the *E. coli* lysate produced the highest yields, but the RITs synthesized in the CHO lysate were up to four times as cytotoxic on CD7-positive Jurkat, HSB-2, and ALL-SIL cells. Furthermore, proteins derived from the microsomal lumen showed a higher toxicity than the RITs that were not translocated [22].

These studies have shown the potential of CFPS for the development of biopharmaceuticals. Considering the advantages of cell-free systems, such as parallelizability, PCR template usage, and the potential for up- and downscaling of the synthesis reaction, a high-throughput screening of various toxic moieties including diverse mutations can be performed (Figure 2). This will allow a faster drug development process. 

## 3. Discussion

Analysis of the structure and mechanism of action of toxins is essential for research and development in the field of toxicology. When studying toxins, diverse aspects need to be considered. The toxin itself must be available; thus, it needs to be isolated from the bacterium, plant, or animal venom that it originates from, or it has to be synthesized synthetically. As the nature of the toxin is to harm certain organisms, this synthesis can be difficult. To date, only a few scientific groups have worked on the synthesis and functional characterization of toxins using cell-free systems. The gained total protein yields within cell-free systems are currently still rather low; thus, the synthesis of protein toxins appears to be more favorable in cell-based bacterial systems. An experimental study comparing the expression of a model toxin in cell-based and cell-free pro- and eukaryotic systems would be beneficial to compare the efficiency of CFPS with that of conventional cell-based methods within one study. In general, the optimization of cell-free systems for toxin synthesis would increase the utilization of CFPS and the availability of published data. The open nature of the cell-free system allows the easy modification of the reaction parameters according to the essential needs of the toxin of interest. With regards to prokaryotic cell-free systems, the supplementation of chaperones has boosted the efficiency of cell-free toxin synthesis [22]. Furthermore, the PURE *E. coli* system offered suitable surroundings for the synthesis of venom toxins [66]. When considering eukaryotic cell-free systems, ER-derived microsomal structures allow the synthesis of functionally active protein, thus representing a crucial and non-negotiable component for cell-free syntheses [27,57]. Therefore, it can be concluded that the synthesis parameters have to be chosen accurately within each cell-free system and for each individual protein.

In 1974, cell-free systems were utilized to aid in the analysis of protein-based toxins for the first time [44]. Over the last decades, CFPS has been established as a useful alternative for studying protein toxins. Nonetheless, these proteins are still of toxic nature, and various toxins are part of the dual-use regulation [113,114]. Therefore, researchers should always consider the possibility of replacing the toxin or toxic moiety with another substance. Additionally, the production of toxins for research purposes is limited to low scales. The scalability of cell-free systems could be an advantage for that matter, as high-throughput screenings (HTS) at scales as low as nL or µL are possible [20]. 

Using CFPS in a parallelized manner to compare the effects of different mutations has been shown before. This can easily be transferred to the analysis of toxins, e.g., for the identification of catalytic centers of a toxin. In cell-free systems, this can be accomplished by adding different templates in parallel syntheses, allowing the screening of diverse proteins, mutants or even combinations of different templates. The addition of different templates is also essential for studying protein complexes and subunit interactions. This has been shown for diverse toxins, such as PFTs or AB_5_ toxins, but also for various membrane proteins and viral particles [52,115,116]. As toxins and membrane proteins oftentimes interact on a cellular level, CFPS might further allow the investigation of interaction partners in an in vitro surrounding.

Prior work has demonstrated the possibility of adapting CFPS to screening technologies such as HTS and the automatization of the process [117,118]. For example, it has been used in a nucleic acid programmable protein array (NAPPA) approach as an array-based system for protein interaction studies [119]. This opportunity presented by CFPS-based screenings could be transferred to toxin blockers/inhibitors. The toxins could be produced in a small scale, and the most efficient inhibitors could be identified with a HTS. This system requires only small amounts of toxins and thus would be in accordance with official regulations. In small-scale productions, toxins and toxin fragments from cell-free reactions could be used as reference material for the establishment of detection assays. For that matter, the synthesizability and purity of the toxin, as well as the acquired total protein yield, must be optimized further. In general, prokaryotic cell-free systems are known for higher protein yields, while eukaryotic systems show more diverse PTM patterns [20,25]. Over the last years, the yields in eukaryotic systems, such as the CHO system, were optimized and can now compete with prokaryotic systems whenever the optimizations are kept in mind [21]. Eukaryotic systems will be more favorable for the synthesis of complex protein toxins, especially animal toxins, as a native folding environment would be more suitable for the synthesis of these proteins. Unfortunately, not all studies were able to synthesize protein toxins in a cell-free manner. Mainly, these were toxins from animal venoms. As described, one of these toxins was the complex, disulfide-bridge-containing U_2_-sicaritoxin-Sdo1a from a spider venom, which could be synthesized only in the *E. coli* PURE system. Even though the *Sf*21 system is known for very efficient disulfide bridge formation [25], it failed to synthesize this toxin [66]. In a different study, 30 different toxins from venom compositions were tested in an *E. coli* PURE system. Strikingly, only four proteins could be synthesized [67]. These data demonstrate the necessity of an adaptable system. Commercially available synthesis kits might not be the ready-to-use option for venom proteins. As the cell-free system is an open system, the synthesis of individual toxins should be adjusted to their individual needs in order to ensure synthesizability as well as a sufficient protein yield.

The synthesized proteins could be used as reference material for the development of diagnostic assays, for antibody and antidote generation and screening, or even for referencing foodborne toxins in nutritional samples. For the screening of toxin blockers/inhibitors, they could even further be evaluated for their potential in pharmaceutical application. The time for the development of such an antidote/blocker would be tremendously reduced, as the toxin synthesis and screening of the binding could be performed within several hours. Unfortunately, not many studies have compared cell-based or naturally extracted toxins with those produced in cell-free reactions. To validate CFPS for further downstream applications, future studies should address this matter. Commercial detection assays in the medical field, as well as in the agricultural and nutritional sectors, should be tested with cell-free synthesized toxins in order to validate whether CFPS could be used in such a manner. In one attempt, it was shown that cell-free synthesized Nhe could be detected in a commercial detection assay for bacterial contamination in food [52]. During the expression of proteins, such as protein toxins, in cells, a purification step is necessary. When using CFPS, this is not mandatory, as endotoxin-free lysates are used [22,25]. This is advantageous, as purification might lead to inactive proteins because of the use of diverse chemicals during such a procedure [120]. 

Furthermore, if an N- or C-terminal purification tag on the protein is used, tertiary structure formation might be disturbed. Bechlars et al. showed that the addition of purification tags altered the functional activity of the PFT TDH [49]. Therefore, a fast initial screening can easily be done without prior purification. For diagnostic or medical approaches, purifications are necessary in the long run.

Screening of toxic moieties for pharmaceutical purposes might be one of the most beneficial applications of CFPS-based toxin research. Fast and efficient screening of targeted toxins is possible. Several studies have demonstrated that the synthesis and downstream characterization of these targeted toxins can be performed in cell-free systems. Nonetheless, at later stages in clinical development, upscaled production is necessary. Hence, nontoxic alternatives that do not require dual-use products should always be taken into consideration.

The work of the last decades has shown the possibilities of cell-free toxin synthesis. Within diverse studies at the beginning of toxin research in cell-free systems, mRNA extracted from animals was used for protein synthesis. While this is a necessary feature for the identification of newly found venoms, the venom extraction can be rather harmful for the animals. For later studies of inhibitors, or even the modification of venomous compounds, e.g., for biopharmaceuticals, CFPS is a beneficial alternative and will reduce animal suffering for research purposes in accordance with the 3R principle [121]. The 3R principle defines the reduction and replacement of animal testing as well as the refinement of experimental setups to lower animal suffering. Synthesizing animal venom in cell-free systems would replace the stressful and painful techniques for extraction from animals. The production of biopharmaceuticals such as antibodies or antibody–drug conjugates can be parallelized and simultaneously screened in in vitro assays. This will allow faster identification of potential drug candidates and lead structures. Hence, this will further lower the number of animal studies, as fewer compounds will have to be tested in in vivo trials.

Scalability, high-throughput screening compatibility, time efficiency, and accordance with the 3R principle are advantages for cell-free toxin synthesis.

## 4. Conclusions

All in all, CFPS is a versatile and inevitable platform technology for the fast and efficient synthesis of protein toxins. CFPS can be used to synthesize, functionally characterize, and even modify protein toxins for downstream applications. Diverse protein toxins have been synthesized in various cell-free systems. Based on the published studies, it can be said that bacterial toxins have been studied the most, while fungal and plant-based toxins have rarely been studied. PFTs can be synthesized in an efficient manner in both pro- and eukaryotic cell-free systems. Other toxin classes, such as RIPs and ADP-ribosyltransferases, need optimized eukaryotic systems. Venom toxins have been studied, but to date, the efficiency of cell-free systems for this large and diverse class of proteins has not yet been fully evolved.

Optimized synthesis and subsequent functional analysis of these toxin classes should be addressed in the future. Furthermore, it will be necessary to enhance this platform for the synthesis of complex proteins, such as those found in animal venoms, and for the synthesis of whole venom mixtures. In that matter, it might be useful to develop novel cell-free protein synthesis platforms derived from other cell types or modify existing lysates, e.g., by integrating synthesis-enhancing proteins, as shown for the integration of the T7 polymerase by using the CRISPR technology [122]. To fully assess the effectiveness of cell-free systems for toxin synthesis, a functional characterization of the toxin’s activity must be assessed after every synthesis. The potential benefits of CFPS for the synthesis of toxins have been demonstrated. Nevertheless, further optimization strategies are necessary to validate cell-free systems for toxin synthesis. Further intense testing will help to strengthen the role of CFPS in this matter.

CFPS and downstream applications allow an interplay of versatile scientific disciplines and will further help to characterize protein toxins, aid in the identification of toxin inhibitors, and facilitate the development of novel pharmaceuticals. Nonetheless, as the basic nature of toxins is harmful, their application should be limited to research aiming to understand their underlying functional mechanisms and subsequently identify inhibitors against toxins; they should never be exploited for dual-use purposes.

## Figures and Tables

**Table 1:** Overview of cell-free synthesized protein toxins.

Protein Toxin	MoA	Cell-Free System	Application/Assay System	Functionality	Year
Bacterial Protein Toxins					
Diphtheria toxin	ADP-ribosyltransferase	*E. coli*	Rabbit skit toxicity	yes	1974 [44]
Heat-stable enterotoxin	Activation of cGMP system	*E. coli*	Protein analytics	yes	1980 [46]
Leukocidin	PFT	*E. coli*	Electrophysiology Hemolysis	yes	2001 [47]
α-hemolysin	PFT	*E. coli*	Pore formation in bilayersFluorescence microscopy	n.a.	2011 [48]
TDH and TDH-related hemolysin	PFT	*E. coli*	HemolysisAgglutination assay Electrophysiology	yes	2013 [49] 2015 [50] 2018 [51]
Non-hemolytic enterotoxin	PFT	CHO	Hemolysis Electrophysiology Cell-based toxicity Fluorescent labeling	yes	2020 [52]
Hemolysin BL	PFT	CHO	Hemolysis Cell-based toxicity	yes	2021 [53]
Streptolysin O toxoid	PFT	Wheat Germ	Modification with ncAA Vaccine development	Antibody production triggered	2022 [54]
Cholera toxin	ADP-ribosyltransferase	CHO*Sf*21	Cell-based toxicity Mutational analysis and modification with ncAA Fluorescent labeling	yes	2022 [55]
Heat-labile enterotoxin	ADP-ribosyltransferase	CHO *Sf*21	Cell-based toxicity Streptavidin modification Fluorescent labeling	yes	2022 [55]
Cytotoxin K	PFT	CHO	Hemolysis Electrophysiology Mutational analysis	yes	2022 [56]
Shiga toxin	RIP	*E. coli* CHO	Cell-based toxicity In vitro protein translation inhibition Comparison of Shiga variants	yes	2024 [57]
Bacteriophage Protein Toxin					
MS2-L toxin	PFT	*E. coli*	Protein assembly analysis	n.a.	2023 [58]
Protein Toxins from Venoms					
Honeybee venom: melittin	PFT	Wheat Germ	Protein analysis Sequence analysis	n.a.	1977 [45] 1978 [59]
Queen bee venom: preprosecapin	Serine-Protease Inhibitor-like Protein	Wheat Germ	mRNA analysis	n.a.	1984 [60]
South African puff adder venom	Cytotoxin, Hemolysin	Wheat Germ Rabbit Reticulocyte	mRNA analysis	n.a.	1985 [61]
Venom kallikrein	Serine Protease	Wheat Germ	Enzymatic substrate hydrolysis	yes	2012 [62]
*Pieris rapae* toxin: pierisin	ADP-ribosyltransferase	*Sf*21	In vitro DNA-ADP-ribosylation Cell-based toxicity Fluorescent labeling On chip synthesis	yes	2011 [63] 2016 [64]
*Bothrops jararaca* precursor of HF3	Metalloproteinase	*E. coli*	Fibrinogen cleavage	yes	2016 [65]
*Hexophtalma dolichocephala* U_2_-sicaritoxin-Sdo1a venom peptide	Knottin	*Sf*21 (no TL) *E. coli* (no TL) *E. coli* PURE	Cell-based toxicity	n.a. n.a. yes	2021 [66]
Comprehensive venom biodiscovery (including 30 different venoms)		*E. coli* PURE	Evaluation of translation efficiency	n.a.	2024 [67]
Targeted Toxins					
Diphtheria toxin-scFv	ADP-ribosyltransferase	Rabbit reticulocyte	Cell-based toxicity	partially	1993 [68]
PE-scFv	ADP-ribosyltransferase	Rabbit reticulocyte	Cell-based toxicity	yes	1993 [68]
Tetanus toxin fragment–scFv fusion	Neurotoxin	*E. coli*	Vaccine for antitumor response	yes	2011 [39]
Dianthin-EGF	RIP	CHO	In vitro protein translation inhibition Cell-based toxicity Tumor targeting	yes	2022 [56]
PE-anti-CD7 scFv	ADP-ribosyltransferase	*E. coli* CHO	Cell-based toxicity	yes	2022 [22]

MoA: mode of action; ADP: Adenosine diphosphate; cGMP: cyclic guanosine monophosphate; TDH: Thermostable direct hemolysin: CHO: Chinese hamster ovary; *Sf*21: *Spodoptera frugiperda*; PFT: pore-forming toxin; RIP: ribosome-inactivating protein; ncAA: noncanonical amino acid; TL: translation; PE: Pseudomonas exotoxin; scFv: single-chain variable fragment; EGF: Epidermal growth factor.

## Data Availability

No new data were created or analyzed in this study. Data sharing is not applicable to this article.
